# Response to ‘Serum level of adiponectin is a surrogate independent biomarker of radiographic disease progression in early rheumatoid arthritis: results from the ESPOIR cohort’– authors’ reply

**DOI:** 10.1186/ar4537

**Published:** 2014-04-10

**Authors:** Eric Toussirot, Gilles Dumoulin

**Affiliations:** 1 University Hospital of Besançon, Clinical Investigation Center for Biotherapy, INSERM CBT-506, FHU INCREASE, Besançon 25000, France; 2 University Hospital of Besançon, Endocrine and Metabolic Biochemistry, Besançon 25000, France; 3 University of Franche Comté, UPRES EA 3920 “Cardiovascular Pathophysiology and Prevention”, SFR FED, 4234 Besançon, France

## 

We read with great interest the recent article in *Arthritis Research & Therapy* in which Meyer and colleagues evaluated different circulating adipokines in patients with recent-onset rheumatoid arthritis (RA) [1]. The authors found that total adiponectin was independently associated with baseline radiographic score and with a change in this score over time. The authors concluded that total adiponectin at the time of diagnosis is a surrogate marker for radiographic progression in early RA.

However, there are some compelling reasons why the role of adiponectin in RA requires further elucidation. The conclusion by Meyer and colleagues should thus be tempered.

First, several studies have investigated circulating concentrations of leptin, adiponectin, visfatin and resistin in RA, showing, in general, elevated levels. However, the link between these adipose products and disease activity remains controversial.

Second, both proinflammatory and anti-inflammatory effects of adiponectin have been reported.

Third, adiponectin exists in various isoforms, monomers and multimers. These different isoforms perform distinct and sometimes counteracting biological functions: low molecular weight adiponectin has been shown to inhibit lipopolysaccharide-mediated IL-6 release and to stimulate IL-10 secretion [2]. Conversely, high molecular weight (HMW) adiponectin induces secretion of IL-6 by monocytes, and increases production of monocyte chemoattractant protein-1 and IL-8 by peripheral blood mononuclear cells and microvascular endothelial cells [3].

Fourth, in a cross-sectional study, we evaluated total adiponectin and HMW adiponectin in patients with established RA and in healthy controls. We found that circulating HMW adiponectin did not differ between patients and controls, whereas total adiponectin was elevated in the RA group. In addition, total adiponectin and HMW adiponectin did not correlate in this series [4].

Fifth, adiponectin has been shown to be associated with disease severity or joint destruction in both cross-sectional and longitudinal studies with conflicting results [1]. Moreover, in a murine collagen-induced arthritis model of RA, total adiponectin was shown to attenuate the severity of arthritis [5].

Finally, studies conducted to date in RA measured exclusively total adiponectin and not its isoforms, and the discrepancies between the findings of these studies may be explained by the distinct biological properties of the different adiponectin isoforms. In this context, no formal conclusion may be drawn about the role of adiponectin in disease activity and severity in RA. Future studies evaluating adiponectin in RA and its relationships with radiographic progression should include assessment of the different adiponectin isoforms.

### Abbreviations

HMW: High molecular weight; IL: Interleukin; RA: Rheumatoid arthritis.

### Competing interests

The author declares that he has no competing interests.
